# *Neisseria gonorrhoeae* FC428 Subclone, Vietnam, 2019–2020

**DOI:** 10.3201/eid2802.211788

**Published:** 2022-02

**Authors:** Trang Minh Trinh, Tam Thi Nguyen, Thanh Viet Le, Trang Thu Nguyen, Dan Thi Ninh, Bao Hac Duong, Minh Van Nguyen, Thomas Kesteman, Lan Thi Pham, H. Rogier van Doorn

**Affiliations:** National Hospital of Dermatology and Venereology, Hanoi, Vietnam (T.M. Trinh, L.T. Pham);; Hanoi Medical University, Hanoi (T.M. Trinh, L.T. Pham);; Oxford University Clinical Research Unit, Hanoi (T. Thi Nguyen, T. Thu Nguyen, T. Kesteman, H.R. van Doorn);; Quadram Institute Bioscience, Norfolk, UK (T.V. Le);; Hospital of Dermatology and Venereology, Ho Chi Minh City, Vietnam (B.H. Duong);; Hospital of Dermatology and Venereology, Da Nang City, Vietnam (M.V. Nguyen);; University of Oxford, Oxford, UK (T. Kesteman, H.R. van Doorn)

**Keywords:** Neisseria gonorrhoeae, gonorrhea, FC428 clone, multidrug resistance, antimicrobial resistance, cephalosporin resistance, bacteria, Vietnam

## Abstract

Among 114 clinical *Neisseria gonorrhoeae* isolates collected in Vietnam during 2019–2020, we detected 15 of subclone sequence type 13871 of the FC428 clonal complex. Fourteen sequence type 13871 isolates with mosaic *penA* allele 60.001 were ceftriaxone or cefixime nonsusceptible, and 3/14 were azithromycin nonsusceptible. Emergence of this subclone threatens treatment effectiveness.

Gonorrhea is a sexually transmitted infection caused by *Neisseria gonorrhoeae*; global incidence is ≈80 million cases/year (*1*,*2*). To treat uncomplicated gonorrhea, the World Health Organization recommends dual therapy with a single-dose extended-spectrum cephalosporin (ESC) (intramuscular ceftriaxone or oral cefixime) and oral azithromycin (*3*). However, *N. gonorrhoeae* resistance to ESCs and azithromycin was recently reported (*4*).

A ceftriaxone-resistant strain (FC428) harboring mutations of mosaic *penA* allele 60.001 (*penA*-60.001) and belonging to sequence type (ST) 1903 was detected in Japan in 2015 (*5*) and has now been reported on all continents (*6*). A subclone of FC428, which also carried a mosaic *penA*-60.001 gene but belonged to ST13871, was detected in Singapore in 2018 (isolate 18DG342) and in France in 2019 (isolate F91) (*7*,*8*). Genomic surveillance of *N. gonorrhoeae* in Vietnam during 2011 and 2015–2016 showed 1%–5% resistance to ceftriaxone that was not associated with *penA*-60.001 (*9*). In 2019–2020, we detected 15 ST13871 isolates related to the FC428 clone in Vietnam. 

## The Study

During June 2019–December 2020, a total of 1,116 *N. gonorrhoeae* isolates were isolated from 6,090 urethral and endocervical swab samples at 3 dermatology and venereology hospitals in Hanoi, Danang, and Ho Chi Minh City, Vietnam. Of these, 427 isolates were sent to a reference laboratory (National Hospital for Tropical Diseases, Hanoi) for antimicrobial susceptibility testing and sequencing. We used disk diffusion (Oxoid, http://www.oxoid.com/uk) to determine susceptibility to penicillin, tetracycline, spectinomycin, ciprofloxacin and an Etest (bioMérieux, https://www.biomerieux.com) for ceftriaxone, cefixime, and azithromycin. We interpreted inhibition zones and MICs according to 2020 Clinical and Laboratory Standards Institute guidelines (*10*). For whole-genome sequencing, we selected 114 isolates (28/104 from Hanoi, 16/56 from Danang, and 70/267 from Ho Chi Minh City) according to reduced susceptibility to ESC/azithromycin (51/114) or epidemiologic features (travel, men having sex with men, contact with sex worker, multiple partners). This study was approved by the Institutional Ethical Review Board of Hanoi Medical University, Hanoi, Vietnam (518/GCN-HDDDNCYSH-DHYHN, 2021 May 17). All participants gave written informed consent.

After extracting DNA with a DNeasy Blood & Tissue kit (QIAGEN, https://www.qiagen.com), we prepared DNA libraries by using Nextera XT library preparation and index kits (Illumina, https://www.illumina.com). We performed sequencing on a MiSeq platform with reagent kit 600 V3 (Illumina). We used fastp version 0.20.0 (GitHub, https://github.com) to filter out low-quality bases with Phred score <30 and to trim off the adapters. We used ARIBA 2.14.6 (GitHub) with a custom database for screening to detect antimicrobial resistance genes**.** We identified multilocus sequence typing (MLST) records from the *Neisseria* typing scheme PubMLST (https://pubmlst.org). We performed de novo assembly on the processed reads by using Shovill version 1.1.0 with SPades version 3.14 (GitHub) as the assembler. We used MOB-suite version 3.0.0 (GitHub) to reconstruct chromosome and plasmids from the assemblies. We identified *N. gonorrhoeae* multi-antigen sequence type (NG-MAST) by using NGMaster version 0.5.5, and we used *N. gonorrhoeae* Sequence Typing for Antimicrobial Resistance (NG-STAR) with pyngSTar (GitHub). We used the closest complete genome of *N. gonorrhoeae* searched by ReferenceSeeker (GitHub) as reference in Snippy 4.6.0 (GitHub) for variant calling. We created the core-genome alignment by using snippy-core with a provided mask of repeated regions and mobile elements. We used Gubbin version 2.3.4 (GitHub) to filter out the recombination in the alignment and fed it into IQTREE2 (GitHub) to reconstruct a maximum-likelihood phylogenetic tree. We used BEAST version 10.4 and TreeAnnotator (https://beast.community) to estimate the time to the most recent common ancestor (tMRCA), and ggtree version 3.0.2 (GitHub) in R (https://www.r-project.org) for visualization. Sequencing data are available from the European Nucleotide Archive (https://www.ebi.ac.uk/ena; project no. PRJEB45627).

Of 114 *N. gonorrhoeae* isolates, 15 were typed by MLST as ST13871 (Table). All patients recovered clinically after receiving 1 dose of intramuscular ceftriaxone (1 g) and oral azithromycin (1 g), although microbiological clearance of *N. gonorrhoeae* was unknown. However, because neither test-of-cure nor pharyngeal testing was performed, persistent asymptomatic infection may have been missed.

**Table:** Epidemiologic and clinical characteristics of patients infected with multidrug-resistant *Neisseria gonorrhoeae* ST13871, Vietnam, 2019–2020*

Variable	No. (%)
Patient sex	
M	13 (87)
F	2 (13)
Place of consultation, year	
Ho Chi Minh City, 2019	3 (20)
Ho Chi Minh City, 2020	9 (60)
Danang, 2020	3 (20)
Hanoi	0
Clinical history	
Previous STIs	0
Co-infection with other STIs	
Syphilis	0
*Chlamydia trachomatis*	0
Sexual history	
Sex partners during past 3 mo	
1	10 (67)
>2	5 (33)
Sexual contact with commercial sex worker	5 (33)
Unprotected sex during most recent intercourse	12 (80)
Men who have sex with men	0
Current treatment with antibiotic	15 (100)
Ceftriaxone, 1g intramuscularly	15 (100)
Azithromycin, 1g orally	10 (67)

*Patient age range (median) 19–56 (31) y. ST, sequence type; STIs, sexually transmitted infections.

Among the 15 ST13871 isolates, NG-MAST based on 2 antigen genes identified 7 as ST7237 and 1 as ST1086; 7 were of unidentified sequence type (*porB* new, *tbpB* 21). In the NG-STAR system, based on 7 resistance genes, we found that 6 isolates were ST233, 1 was ST345, 1 was ST1133; sequence types were unknown for 7 (Appendix). For 1 isolate from Vietnam, the MLST, NG-MAST, and NG-STAR typing was identical to that of the 2 strains reported from Singapore and France/Cambodia (Appendix).

All ST13871 isolates were resistant to ciprofloxacin, nonsusceptible to penicillin and tetracycline, but susceptible to spectinomycin. For 14 isolates, susceptibility to ESCs was reduced (MIC ranges: cefixime 0.5–1.5 mg/L, ceftriaxone 0.38–0.75 mg/L) and 3 were nonsusceptible to azithromycin (MIC 1.5 mg/L), thus presenting an extensively drug-resistant (XDR) pattern (*11*) (Appendix).

Most ST13871 isolates harbored resistance genes, including *ponA*:L421P, the plasmid-mediated *blaTEM* 135, the *mtrR* promoter −35Adel mutation causing overexpression of the MtrCDE efflux pump, *rpsJ*:V57M associated with tetracycline resistance, or changes in *penB* (G120K, A121D) associated with decreased influx of porin channel PorB1b. The mosaic *penA*-60.001, associated with resistance to ESCs (*5*), was found in 14 of 15 isolates, all nonsusceptible to >1 ESC (Appendix). One ST13871 isolate carrying *penA*-43.002 gene was susceptible to ESCs.

In all 15 isolates, we found mutations in *gyrA* and *parC* genes, conferring resistance to ciprofloxacin, including *gyrA*: S91F/G95A and *parC*: S87R, and 2 mutations (*parC*: V596I, L479F) not previously reported. We found no plasmidborne *tetM* causing resistance to tetracycline and no other gene except for *mtrR* promoter −35Adel conferring resistance to azithromycin.

According to time-scaled Bayesian phylogeny of 17 ST13871 sequences from Vietnam (n = 15), France (n = 1), and Singapore (n = 1) (Figure), samples clustered into 3 distinct clades. Clade 1a contained the 2 previously reported ST13871 isolates from Singapore and France/Cambodia, as well as 3 isolates from this study, including the one sharing the same typing as the international isolates. Median tMRCA of this clade was calculated as March 2017 (95% highest posterior density [HPD] April 2014–February 2018), of clade 1b as October 2018 (95% HPD August 2016–September 2019), and of clade 2 as September 2017 (95% HPD April 2013–May 2019). Estimated median tMRCA for the 17 ST13871 isolates was September 2014. One clade 1b isolate came from a patient who reported having had sexual contact in Laos 1 week before diagnosis. Two of the XDR isolates belonged to clade 2 but were not closely related.

**Figure:**
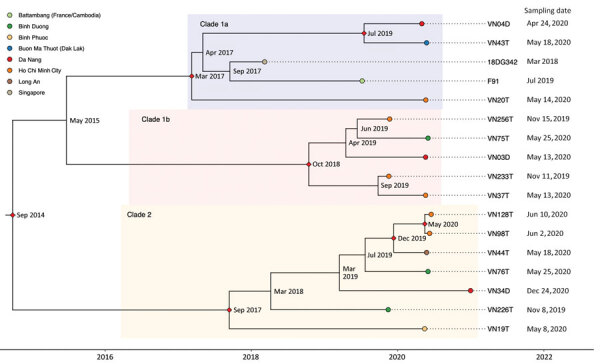
Time-scaled Bayesian maximum clade credibility phylogenetic tree of *Neisseria gonorrhoeae* ST13871 (17 isolates) with date of collection and location of collected isolates, Vietnam, 2019–2020. Red diamonds show posterior probability >90%; internal node labels show estimated time to most recent common ancestor.

## Conclusions

We detected the globally disseminated FC428-related resistant *N. gonorrhoeae* clone in Vietnam in 2019–2020. Among 114 *N. gonorrhoeae* isolates collected, 15 were ST13871 and 14 were related to the FC428 clone by harboring the mosaic *penA–*60.001 gene conferring resistance to ESCs. The ceftriaxone MICs for these 14 isolates were similar to those for globally reported FC428 isolates, but cefixime MICs were lower (*6*,*12*). We found 3 XDR ST13871 isolates, nonsusceptible to azithromycin and ESCs but susceptible to spectinomycin. Resistance determinants to other antimicrobial drugs in all isolates from Vietnam were similar to those of the FC428 clone (*5*,*7*,*8*,*13*,*14*).

Our phylogenetic analysis showed that all 17 ST13871 isolates arose from the rooted FC428 strain and were distributed into 3 clades with a common ancestor estimated in 2014, consistent with estimates of other FC428-like isolates (*13*). These results suggest that ST13871 has been circulating in Southeast Asia for several years.

Emergence of multidrug-resistant FC428 subclone (ST13871) in Vietnam possibly threatens effectiveness of the current presumptive treatment. Therefore, regular monitoring of antimicrobial drug susceptibility of *N. gonorrhoeae* is necessary. Controlling the spread of resistant *N. gonorrhoeae* may be enhanced by follow-up visits, postrecovery culturing, and partner counseling.

Appendix Time-scaled Bayesian maximum clade credibility phylogenetic tree of *Neisseria gonorrhoeae* ST13871, Vietnam, 2019–2020.

## References

[1] Newman L, Rowley J, Vander Hoorn S, Wijesooriya NS, Unemo M, Low N, et al. Global estimates of the prevalence and incidence of four curable sexually transmitted infections in 2012 based on systematic review and global reporting. PLoS One. 2015;10:e0143304. 10.1371/journal.pone.0143304 26646541 PMC4672879

[2] Rowley J, Vander Hoorn S, Korenromp E, Low N, Unemo M, Abu-Raddad LJ, et al. Chlamydia, gonorrhoea, trichomoniasis and syphilis: global prevalence and incidence estimates, 2016. Bull World Health Organ. 2019;97:548–562P. 10.2471/BLT.18.228486 31384073 PMC6653813

[3] World Health Organization. WHO guidelines for the treatment of *Neisseria gonorrhoeae*. 2016 [cited 2021 Apr 3]. https://www.who.int/reproductivehealth/publications/rtis/gonorrhoea-treatment-guidelines/en/27512795

[4] Unemo M, Shafer WM. Antimicrobial resistance in *Neisseria gonorrhoeae* in the 21st century: past, evolution, and future. Clin Microbiol Rev. 2014;27:587–613. 10.1128/CMR.00010-14 24982323 PMC4135894

[5] Nakayama S, Shimuta K, Furubayashi K, Kawahata T, Unemo M, Ohnishi M. New ceftriaxone-and multidrug-resistant *Neisseria gonorrhoeae* strain with a novel mosaic *penA* gene isolated in Japan. Antimicrob Agents Chemother. 2016;60:4339–41. 10.1128/AAC.00504-16 27067334 PMC4914677

[6] Chen S-C, Yuan L-F, Zhu X-Y, van der Veen S, Yin Y-P. Sustained transmission of the ceftriaxone-resistant *Neisseria gonorrhoeae* FC428 clone in China. J Antimicrob Chemother. 2020;75:2499–502. 10.1093/jac/dkaa196 32473014

[7] Poncin T, Merimeche M, Braille A, Mainardis M, Bebear C, Jacquier H, et al. Two cases of multidrug-resistant *Neisseria gonorrhoeae* related to travel in south-eastern Asia, France, June 2019. Euro Surveill. 2019;24:1900528. 10.2807/1560-7917.ES.2019.24.36.1900528 31507264 PMC6737829

[8] Ko KKK, Chio MTW, Goh SS, Tan AL, Koh TH, Abdul Rahman NB. First case of ceftriaxone-resistant multidrug-resistant *Neisseria gonorrhoeae* in Singapore. Antimicrob Agents Chemother. 2019;63:e02624–18. 10.1128/AAC.02624-18 30858209 PMC6496044

[9] Lan PT, Golparian D, Ringlander J, Van Hung L, Van Thuong N, Unemo M. Genomic analysis and antimicrobial resistance of *Neisseria gonorrhoeae* isolates from Vietnam in 2011 and 2015-16. J Antimicrob Chemother. 2020;75:1432–8. 10.1093/jac/dkaa040 32068837 PMC7382555

[10] Clinical and Laboratory Standard Institute. Performance standards for antimicrobial susceptibility testing, 30th edition (M100). Wayne (PA): The Institute; 2020. 10.1128/JCM.00213-21 PMC8601225 34550809

[11] Magiorakos AP, Srinivasan A, Carey RB, Carmeli Y, Falagas ME, Giske CG, et al. Multidrug-resistant, extensively drug-resistant and pandrug-resistant bacteria: an international expert proposal for interim standard definitions for acquired resistance. Clin Microbiol Infect. 2012;18:268–81. 10.1111/j.1469-0691.2011.03570.x 21793988

[12] Yan J, Chen Y, Yang F, Ling X, Jiang S, Zhao F, et al. High percentage of the ceftriaxone-resistant *Neisseria gonorrhoeae* FC428 clone among isolates from a single hospital in Hangzhou, China. J Antimicrob Chemother. 2021;76:936–9. 10.1093/jac/dkaa526 33406237

[13] Eyre DW, Town K, Street T, Barker L, Sanderson N, Cole MJ, et al. Detection in the United Kingdom of the *Neisseria gonorrhoeae* FC428 clone, with ceftriaxone resistance and intermediate resistance to azithromycin, October to December 2018. Euro Surveill. 2019;24:1900147. 10.2807/1560-7917.ES.2019.24.10.1900147 30862336 PMC6415501

[14] Lahra MM, Martin I, Demczuk W, Jennison AV, Lee K-I, Nakayama S-I, et al. Cooperative recognition of internationally disseminated ceftriaxone-resistant *Neisseria gonorrhoeae* strain. Emerg Infect Dis. 2018;24:735–40. 10.3201/eid2404.171873 29553335 PMC5875269

