# Supplementary material for: Neisseria gonorrhoeae FC428 Subclone, Vietnam, 2019–2020
Source: Emerg Infect Dis. 2022 Feb;28(2):432–5. doi: 10.3201/eid2802.211788 (PMC8798686; doi:10.3201/eid2802.211788)
Supplement: Appendix — Time-scaled Bayesian maximum clade credibility phylogenetic tree of Neisseria gonorrhoeae ST13871, Vietnam, 2019–2020. [file 21-1788-Techapp-s1.pdf]

Article DOI: https://doi.org/10.3201/eid2802.211788

Neisseria gonorrhoeae FC428 Subclone, Vietnam, 2019–2020

Appendix

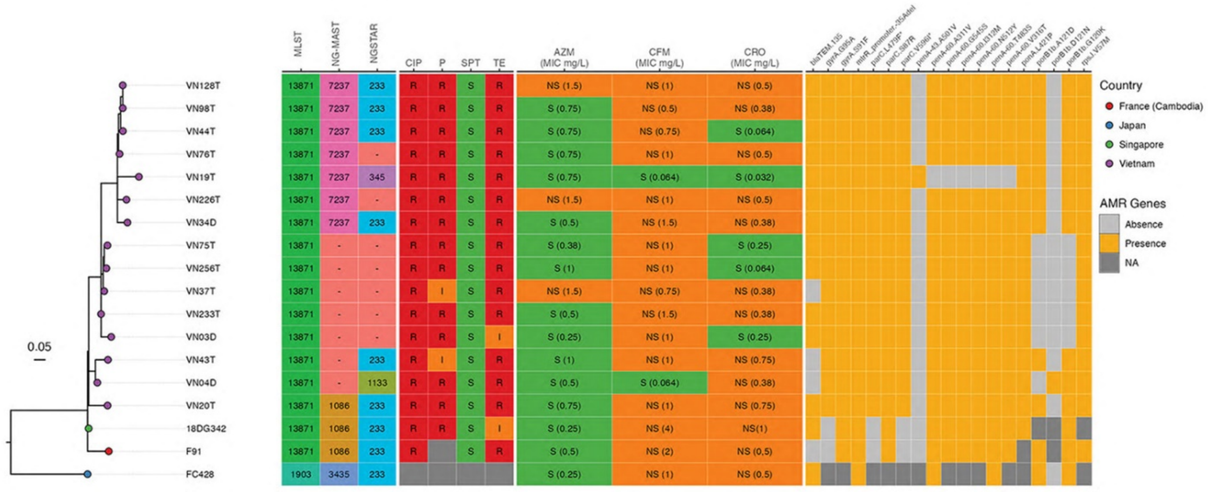

phylogenetic tree of *Neisseria gonorrhoeae* ST13871 samples including 15 isolates from Vietnam during 2019 – 2020, 1 from Singapore (18DG342), and 1 from France (F91), the FC428 strain was used as an outgroup. Sequence types from the MLST, NG-MAST, NGSTAR database; phenotypes and AMR determinants are displayed. Sequence types that have not yet been assigned with a number are indicated as a dash. The phenotypes include Resistance (R), Susceptible (S), Intermediate (I) and Non-susceptible (NS). AMR determinants are antimicrobial resistance gene and their amino acid changes; asterisk symbol (*) refers to a novel mutation. NA (not available) represents phenotypes or resistance genes that were not reported. CIP: ciprofloxacin, P: penicillin, SPT: spectinomycin, TE: tetracycline, AZM: azithromycin, CFM: cefixime, CRO: ceftriaxone.
